# Supplementary material for: Phase 1 dose expansion and biomarker study assessing first-in-class tumor microenvironment modulator VT1021 in patients with advanced solid tumors
Source: Commun Med (Lond). 2024 May 21;4:95. doi: 10.1038/s43856-024-00520-z (PMC11109328; doi:10.1038/s43856-024-00520-z)
Supplement: Supplementary file 2 — Supplementary Information [file 43856_2024_520_MOESM2_ESM.docx]

Supplementary Materials for

**Phase 1 dose expansion and biomarker study assessing first-in-class tumor microenvironment modulator VT1021 in patients with advanced solid tumors**

Jian Jenny Chen1, *, Melanie Y. Vincent1, Dale Shepard2, David Peereboom2, Devalingam Mahalingam3, James Battiste4, Manish R. Patel5, Dejan Juric6, Patrick Y. Wen7, Andrea Bullock8, Jennifer Eva Selfridge9, Shubham Pant10, Joyce Liu7, Wendy Li1, Susanne Fyfe1, Suming Wang1, Victor Zota1, James Mahoney1, Randolph S. Watnick11, Michael Cieslewicz1 and Jing Watnick1, *

*Corresponding authors' emails:

J.C. (jenny.chen@vigeotx.com) and J.W. (jing.watnick@vigeotx.com)

This PDF file includes Supplementary Fig. 1

**Supplementary Figure 1**

**a**


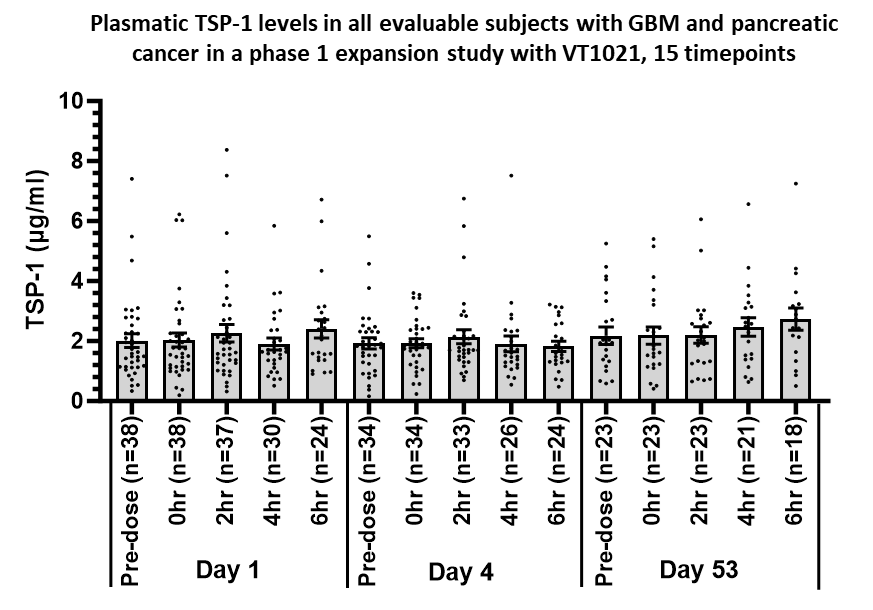


**b**


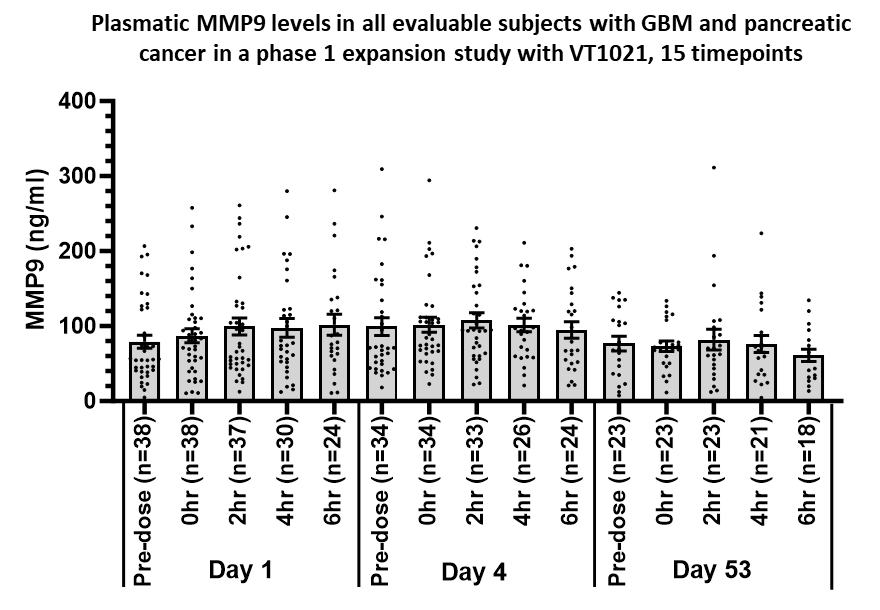


**
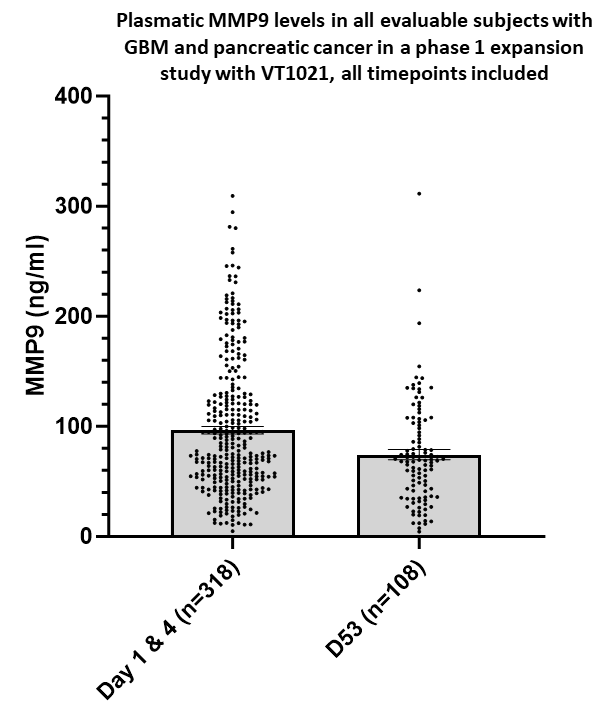

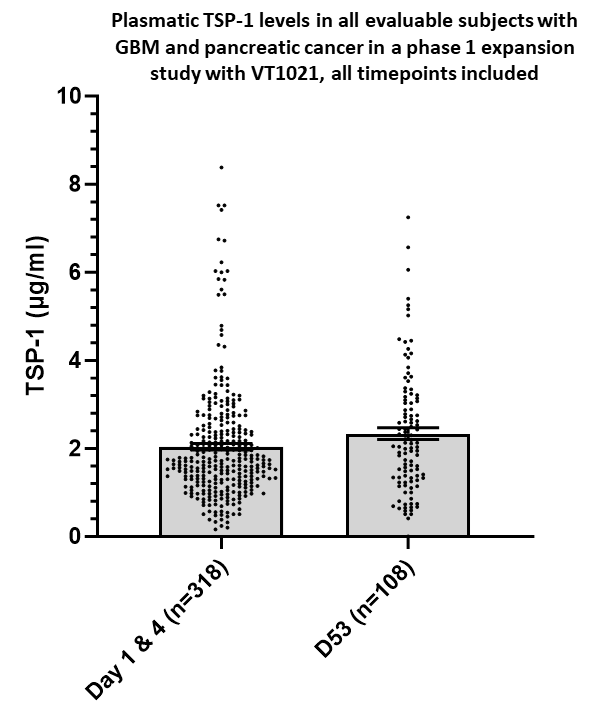
c** **d**

**Supplementary Figure 1.** **Plasmatic TSP-1 and MMP9 levels in all evaluable patients with GBM or pancreatic cancer.** a) TSP-1 levels across 15 timepoints. b) MMP9 levels across 15 timepoints. c) Up-regulation of TSP-1 after 50 days of treatment with VT1021. TSP-1 data points in Day 1 and Day 4 were grouped (mean value = 2.04 µg/ml) and compared to TSP-1 data points in Day 53 (mean value = 2.36 µg/ml). d) Reduction of MMP9 after 50 days of treatment with VT1021. MMP9 data points in Day 1 and Day 4 were grouped (mean value = 96.5 ng/ml) and compared to MMP9 data points in Day 53 (mean value = 74.3 ng/ml). The graphs were plotted with the mean values, error bars indicate SEM, n refers to the number of data points.
