# Supplementary material for: Phase 1 dose expansion and biomarker study assessing first-in-class tumor microenvironment modulator VT1021 in patients with advanced solid tumors
Source: Commun Med (Lond). 2024 May 21;4:95. doi: 10.1038/s43856-024-00520-z (PMC11109328; doi:10.1038/s43856-024-00520-z)
Supplement: Supplementary file 3 — Description of Additional Supplementary Files [file 43856_2024_520_MOESM3_ESM.pdf]

Description of Additional Supplementary Files

File name: Supplementary Data 1
Description: Numerical source data used to create Fig. 1b in the manuscript.

File name: Supplementary Data 2
Description: Numerical source data used to create Fig. 2 in the manuscript.

File name: Supplementary Data 3
Description: Numerical source data used to create Fig. 3a in the manuscript.

File name: Supplementary Data 4
Description: Numerical source data used to create Fig. 4a, 4c, 4d, 4f, and 4g in the manuscript.

File name: Supplementary Data 5
Description: Numerical source data used to create Fig. 5a and 5c in the manuscript.
